# Light-Patterned Current Generation in a Droplet Bilayer Array

**DOI:** 10.1038/srep46585

**Published:** 2017-04-18

**Authors:** Vanessa Restrepo Schild, Michael J. Booth, Stuart J. Box, Sam N. Olof, Kozhinjampara R. Mahendran, Hagan Bayley

**Affiliations:** 1 Chemistry Research Laboratory, University of Oxford, 12 Mansfield Road, Oxford, OX1 3TA, UK

## Abstract

We have created a 4 × 4 droplet bilayer array comprising light-activatable aqueous droplet bio-pixels. Aqueous droplets containing bacteriorhodopsin (bR), a light-driven proton pump, were arranged on a common hydrogel surface in lipid-containing oil. A separate lipid bilayer formed at the interface between each droplet and the hydrogel; each bilayer then incorporated bR. Electrodes in each droplet simultaneously measured the light-driven proton-pumping activities of each bio-pixel. The 4 × 4 array derived by this bottom-up synthetic biology approach can detect grey-scale images and patterns of light moving across the device, which are transduced as electrical current generated in each bio-pixel. We propose that synthetic biological light-activatable arrays, produced with soft materials, might be interfaced with living tissues to stimulate neuronal pathways.

Biological light-sensors range from the high-resolution light-sensing electrical arrays in the retina of the eye^1^ to the simple eye-spot apparatus found in green algae^2^. The retina comprises an array of photoreceptor cells^3^, containing stacks of membrane proteins, the rhodopsins^4^. These biological arrays convert light into an electrical signal^1^. It is of interest to develop soft material arrays that generate patterned current signals by a bottom-up synthetic biological approach.

We have previously used hydrogels as soft materials to build sensing arrays^5^. Bilayers were formed between several hydrogel pillars and a hydrogel surface, which allowed the simultaneous optical recording of currents from individual protein nanopores confined to each pillar. Furthermore, we have previously made a single light-sensing protein-based droplet pixel (bio-pixel) from aqueous droplets-in-oil, containing bacteriorhodopsin (bR) from *Halobacterium salinarum*^6^. bR is a light-driven pump, responding to green light (absorbance maximum of 570 nm), which generates current from the movement of charge, in the form of protons, across membranes^7^,^8^,^9^.

Here, we present a 4 × 4 array of bio-pixels, comprising bR-containing droplets arranged on a single common hydrogel structure. The hydrogel acts both as a scaffold for the array and as a medium for ion transport. Each droplet is interfaced with the hydrogel through a lipid bilayer, into which the bR protein inserts. Protons are pumped from the illuminated droplets into the hydrogel, generating a current across the lipid bilayer, which was converted to an electrical current, amplified and measured. When a light pattern impinges on the array, the currents are recorded simultaneously from each bio-pixel, and represent the original pattern. We have taken inspiration from over 20 years of pioneering work from several groups, which have prototyped bioelectronic imaging devices with bR^10^,^11^,^12^,^13^,^14^, by depositing the protein as films on electrode surfaces. However, in our approach, the structure of the array is a soft material containing droplets interfaced through bilayers to a hydrogel, which allow us to detect patterns of light through current generation. Our droplet array can be regarded as a single device in which a light sensor and a current generator are integrated.

## Results

### Developing a bR bio-pixel

**Figure 1:**
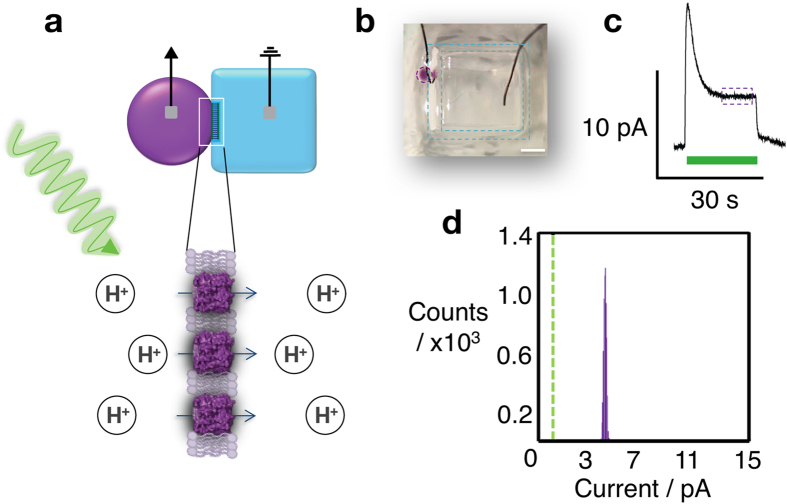
A light-activatable droplet hydrogel bilayer pixel. (**a**) Schematic of a light-activatable droplet hydrogel bilayer (DHB) bio-pixel. In a lipid-in-oil solution, a bR-containing droplet (purple) forms a bilayer with a hydrogel cube (blue). (**b**) Image of a bio-pixel according to the design in (**a**). The bR proteins inserted vectorially into the bilayer and pumped protons across the membrane upon illumination with green light as shown in (**a**). The current across the membrane was measured with Ag/AgCl electrodes inserted in the droplet and the hydrogel. (**c**) Ionic current activation occurred only upon illumination. The purple box indicates the steady-state current. (**d**) All points histogram of the steady-state current from (**c**). A bio-pixel was considered ON when the steady-state current was above a 1 pA cutoff (vertical dashed green line). Scale bar 1.5 mm.

Lipid monolayers form on the surfaces of aqueous droplets and hydrogels in lipid-containing oil. Two droplets can be brought together to form a droplet-interface-bilayer (DIB)^6^, or a single droplet can be contacted with a hydrogel surface to form a droplet-hydrogel-bilayer (DHB)^15^. We have previously generated a light-driven bio-pixel, by incorporating the light-activated proton pump bR into DIBs^6^. In the present work, to allow the development of a droplet array, we required the use of a hydrogel to act as a scaffold, both to position the droplets and form bilayers with them. To this end, we first developed a DHB bio-pixel from a bR-containing droplet and an agarose gel. Our bR suspension was prepared from the purple membrane of *H. salinarum* by using detergent and sonication to prevent aggregation. We observed, no current generation when purple membrane was used without treatment with detergent. The sample was pelleted and resuspended, in a buffer/salt solution, to concentrate the bR before use. The resulting bR suspension was pipetted into a mixture of hexadecane and silicone oil (1:1) containing the lipid 1,2-diphytanoyl-sn-glycero-phosphatidylcholine (DPhPC) to create a 200 nL bR-containing aqueous droplet encapsulated within a lipid monolayer. Bilayers were then formed with a bR-free droplet (DIB) or a hydrogel (DHB) and illuminated at 560 nm. Current responses from the bio-pixels were recorded with Ag/AgCl electrodes (Figs 1 and S1), producing currents similar to those previously reported in different systems^16^,^17^. We designated an arbitrary cut-off current of 1 pA to give each bio-pixel a binary response. Below 1 pA, a pixel is considered to be ‘OFF’, and above 1 pA, a pixel is considered to be ‘ON’ (Fig. 1d).

When the positions of the ground and recording electrodes were switched, the sign of the current reversed, indicating that the observed current corresponds to the directional movement of charge across the bilayer (Fig. S2). In *H. salinarum*, bR pumps protons from its C-terminal face, inside the cell, to the N-terminal face, outside the cell^18^. In our experiments, positive charge moved into the hydrogel, which indicates that bR inserted into the bilayer predominantly N-terminus first. When no bR was present in the bio-pixels, no current was generated during illumination, indicating that artifacts arising from photochemistry at the Ag/AgCl electrodes did not occur (Fig. S3).

### Prototype droplet array

Previously, our group developed a 4 × 4 array for screening ion channels and pores^19^, which employed individual droplets held in electrode-containing poly(methyl methacrylate) (PMMA) wells. A mobile droplet, attached to an electrode on a micromanipulator, was brought into contact with each of the droplets in turn and a membrane current recorded.

As a proof of concept for our droplet array, a prototype 4 × 4 array of bR DIB bio-pixels was made in a similar manner. Following the previous example, currents from each pixel were read one by one with a mobile buffer droplet (Fig. S4). By examining the current outputs from each bio-pixel, we determined the shape of a light beam incident on the array (Fig. S4h), suggesting that patterned current generation stimulated by a shaped light beam would be possible with a further advanced device, which employed simultaneous monitoring of the bio-pixels.

### Droplet-hydrogel array

**Figure 2:**
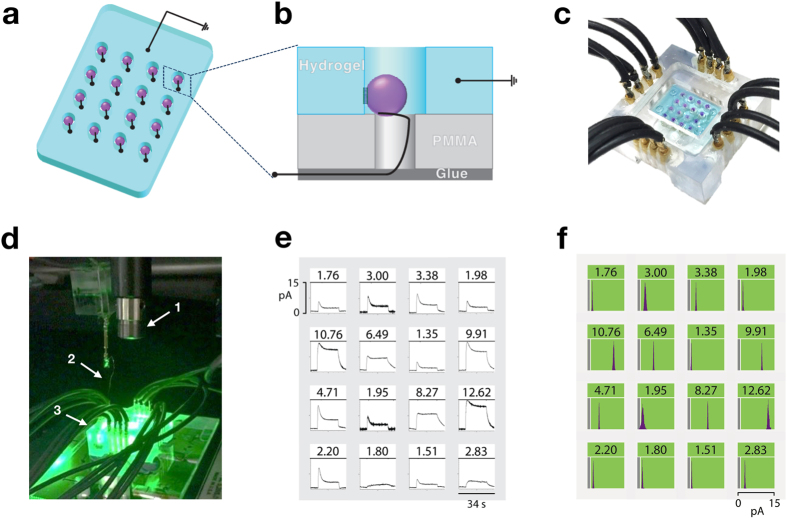
A light-activatable 4 × 4 droplet array. (**a**) Schematic of a 4 × 4 droplet array. Sixteen bR droplets were patterned within a hydrogel structure formed from agarose, in a 4 × 4 array. Each droplet and the hydrogel were connected to an external circuit with Ag/AgCl electrodes. (**b**) Cross section of a single DHB bio-pixel from (**a**). A ground electrode was embedded in the hydrogel, while a recording electrode was inserted into the bR droplet. Each recording electrode was located within a cavity, so that it did not make contact with the hydrogel. (**c**) Image of the 4 × 4 droplet array according to the design in (**a,b**). (**d**) Illumination of the droplet array from above. (1) A fiber-coupled LED (560 nm) illuminates the droplet array. (2) The common ground electrode enters from above and inserts into the hydrogel. (3) Connections from the droplet array to a multichannel amplifier. (**e**) Simultaneous current traces recorded from each bio-pixel of an illuminated 4 × 4 droplet array, with the steady-state mean current values displayed at the top of each trace. (**f**) Distributions of the steady-state current measurements from each pixel of a fully illuminated droplet array (the vertical white line represents the 1 pA cut-off, data below 1 pA is shown in the grey box). All the bio-pixels generated a current above the cut-off.

Therefore, we sought to develop a droplet array in which all the bio-pixels were part of a single soft structure (Fig. 2). This would allow us to use a common ground electrode and detect each bio-pixel individually and simultaneously. Sixteen bR droplets were patterned in a 4 × 4 array onto a hydrogel structure formed from agarose (Figs 2a, S5, S6 and S7). To form the structure, a melted agarose solution was injected into a PMMA chamber (Figs S5 and S6) with a removable lid (Fig. S7a–d), which contained cylindrical PMMA columns to shield the Ag/AgCl electrodes. Once the agarose had gelled, the lid was removed to uncover a rectangular block with a 4 × 4 array of cylindrical cavities (Fig. S7e–g). A Ag/AgCl recording electrode was situated at the bottom of each cavity, while a common ground electrode was inserted into the hydrogel (Fig. S7h,i). The electrodes were connected to a 16-channel amplifier. A solution of 14 mM DPhPC in hexadecane and silicone oil (1:1) was introduced to produce a lipid monolayer on the surface of the hydrogel. bR-containing droplets were pre-incubated in a separate chamber in the same lipid-in-oil solution, so that each became encapsulated in a lipid monolayer. One droplet was then introduced into each of the cylindrical columns, where it was penetrated by a recording electrode and formed a bilayer with the hydrogel structure (Fig. 2b).

The currents from all sixteen bR-containing DHB bio-pixels were recorded simultaneously with a multichannel amplifier during illumination of the hydrogel array from above with a 560 nm LED (Fig. 2d). The current measurements from each bio-pixel were analysed with custom software, which filtered the data and corrected the baselines of the traces (Fig. S8a). The data for each bio-pixel are displayed on a 4 × 4 output grid in three different ways: (1) as current over time graphs (Fig. 2e); (2) as histograms of the steady-state current (Fig. 2f); and (3) as green and grey pixel representations based on whether the current is above (green) or below (grey) the 1 pA arbitrary steady-state-current cut-off (Fig. S8b).

The bio-pixels exhibited a current above the cut-off only when bR droplets were present in the cavities of the hydrogel array and when the device was illuminated (Fig. S9). As expected, bilayer capacitance had an effect on the current profile^20^, which was manifested in different current waveforms from each bio-pixel (Figs 2e and S10) due to variation in the contact areas between the droplets and the hydrogel. No matter the profiles of the currents from bilayers of different capacitance, all bio-pixels generated more current than the 1 pA steady-state cut-off, and so it was possible to determine whether or not a bio-pixel was illuminated.

### Image Detection

**Figure 3:**
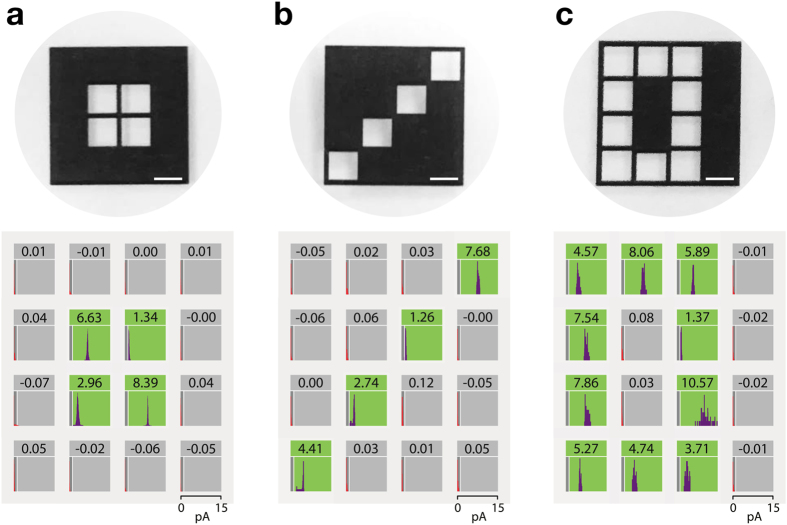
Static shape recognition with the droplet array. (**a**) A photomask of a 2 × 2 square shape was printed on a transparency foil and placed on top of the droplet array. The square image was identified by the droplet array when it was illuminated through the photomask. Mean steady-state bio-pixel currents above the 1 pA threshold reveal the location and shape of the square image. Bio-pixels showing signals above the cut-off are shown with a green background and those with signals below the cut-off have a grey background. (**b**) Recognition of a diagonal line. (**c**) Recognition of the letter O. Scale bars represent 2 mm.

To demonstrate patterned current generation in our device, we first used it to identify static shapes of light impinging on the array. Photomasks were prepared that projected different images onto the bio-pixels (Fig. S11a). The photomasks were placed on top of the droplet array (Fig. S11c) and illuminated from above while the electrical activity in each bio-pixel was monitored. Static images, produced by different photomasks, were identified through the current generated at each of the bio-pixels (Figs 3 and S12).

**Figure 4:**
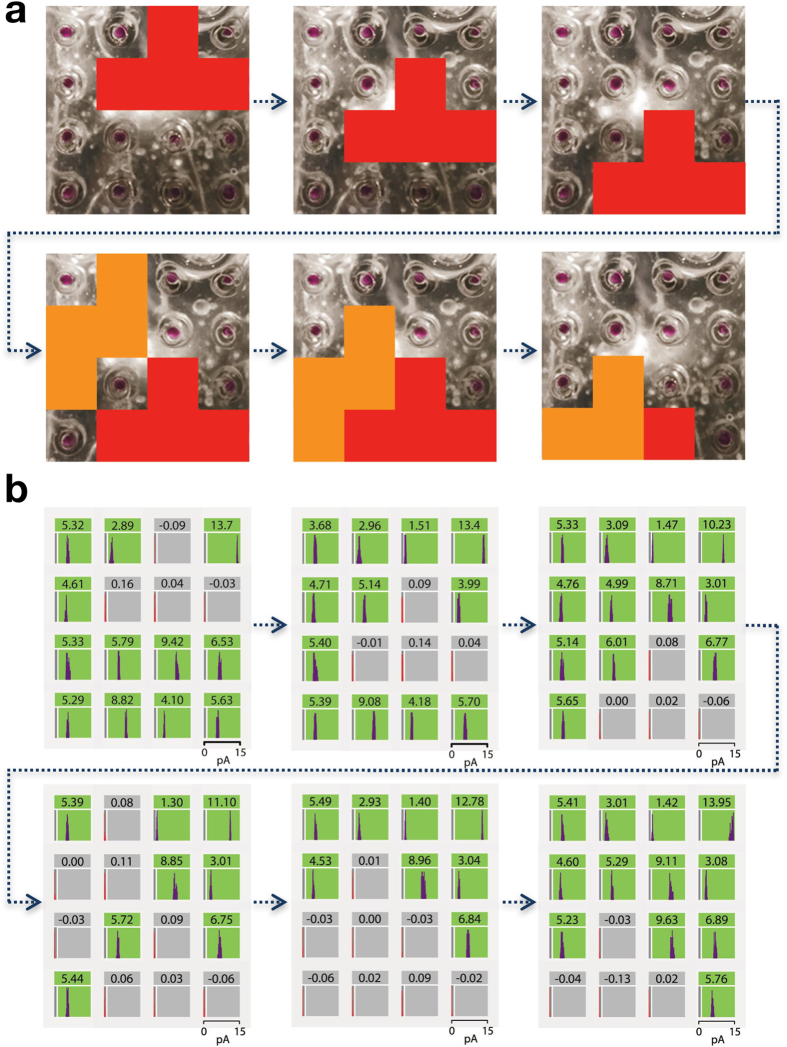
Detecting moving images with the droplet array. (**a**) Two Tetris-based shapes moving down the face of the droplet array one pixel at a time. When the bottom row was completed, both the Tetris blocks were moved down together by one pixel. (**b**) The shapes were revealed by the currents in the active bio-pixels (green). Selected frames are shown here (all frames are shown in Fig. S13).

We next sought to detect moving images with the droplet array. To do this we used 2 × 3 photomasks, which were moved across the surface of the array while the current generation at each bio-pixel was monitored (Fig. 4). These photomasks represented two different Tetris-based shapes (Fig. S11b). Initially, a single shape was detected as it moved across the device, followed by a second shape, culminating in the detection of both shapes moving together after the bottom row had been fully occupied (Figs 4, S13 and Supplementary video). Therefore, we have demonstrated that our droplet array can detect moving shapes, through the light-dependent current patterns generated from a 4 × 4 array of bio-pixels made of soft materials.

### Detecting Greyscale

**Figure 5:**
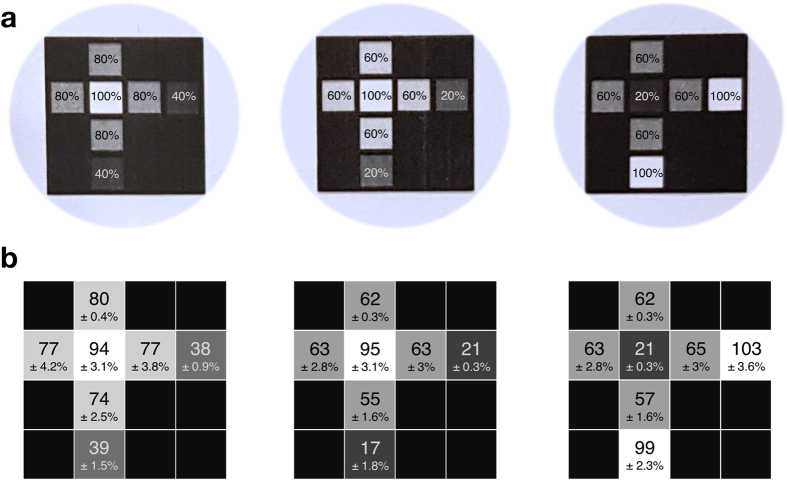
Detecting grey-scale images. (**a**) Photomasks of three grey-scale cross images, containing transparency values from 20–100%. (**b**) Following calibration of the bio-pixels (as shown in Fig. S14), the grey scale cross images were placed on top of the array and then illuminated. The current output of each bio-pixel was converted to a transparency value, based on the calibration. Calculated transparency values and their standard deviations are shown for each bio-pixel.

Following the detection of black and white images using the 1 pA cut off, we investigated the detection of grey scale images. To this end, we produced photomasks of a cross image, made up of different areas of transparency, from 0–100% (Fig. 5a). As current generated from each bio-pixel at the same light intensity varied across the array, because the amount of protein incorporated into each bilayer varied, we calibrated the bio-pixels by using photomasks of 0, 25, 50, 75 and 100% transparency placed on top of the array. Plots of transparency against current output were linear for each bio-pixel (Fig. S14), which allowed the determination of unknown transparency values from current outputs. Following this calibration, the grey-scale cross images were placed over the array and illuminated (Fig. 5b and Tables S1–3) and the transparency values for each bio-pixel established. We reliably detected a wide range of transparency values from multiple grey-scale images.

## Discussion

Here, we have developed a DHB bio-pixel, comprising a bR-containing aqueous droplet interfaced with an agarose hydrogel, which can detect light through the generation of current across the bilayer. These DHB bio-pixels were elaborated into a 4 × 4 array of bR-containing droplets on a common hydrogel scaffold, generating a single soft ‘retina’. The device generated current at each illuminated bio-pixel, individually and simultaneously. By measuring the light-activatable generation of current patterns across our droplet array, we could detect both static and moving images. Furthermore, by calibrating the bio-pixels we were able to detect grey-scale images. As these arrays are made entirely of soft materials, it will be possible to transfer them to aqueous media to allow interaction with living cells. For example, patterned current generation might be used for the targeted stimulation of neuronal pathways.

## Methods

### Buffer Preparation

We used two different buffer solutions throughout. Buffer A contained 10 mM HEPES (Sigma) and 100 mM NaCl (Sigma) at pH 7.5 (titrated with NaOH) and Buffer B contained 100 mM MES (Sigma) and 100 mM NaCl (Sigma) at pH 6.5 (titrated with NaOH). Double-distilled ‘ultrapure’ water (Millipore, Milli-Q: 18.2 MΩ cm) was used throughout.

### Preparation and Gel Analysis of bR

Purple membrane (PM) from *Halobacterium salinarum* contains bacteriorhodopsin (bR) in the form of two-dimensional crystals. Lyophilized PM from *H. salinarum* strain S9 (1 mg protein; Sigma, B0184) was suspended in 40 μL of Buffer B containing 0.01% (w/v) of the detergent n-dodecyl-β-D-maltoside (DDM). The suspension was sonicated in a bath for 30 min at 20 °C (Branson 2800, Cleanosonic), diluted 10-fold in Buffer B and aliquoted into 1.5 mL vials and stored at −80 °C. Prior to use, the bR suspension was centrifuged for 5 seconds and as much supernatant was removed as possible, without disturbing the pellet. The pellet was used to prepare stock solutions of concentrated bR in Buffer B, which were sonicated for 15 min and homogenised (Vortex-Genie 2, Scientific Industries) before use for bR droplets. The protein concentration was ~4 mg mL^−1^ (Nanodrop 1000, assuming an extinction coefficient of 75 000 M^−1^ cm^−1^ at 280 nm)^21^. Portions of the bR suspension were run on a denaturing PAGE gel (Mini-Protean^®^ TGX™ 10% Precast gel) with SDS-containing running buffer to confirm the purity of the protein monomer. Only a single band was visible at 27 kDa based on the Precision Plus Protein Dual Colour Standards (Bio-Rad).

### Preparation of Ag/AgCl Electrodes

Silver wires (100 μm diameter, Sigma) were soldered into male crimp-terminals (RS Components). The tips of the wires were incubated in sodium hypochlorite, NaClO (10% active chlorine, Sigma) for 1 h to form Ag/AgCl electrodes. The tips of the electrodes were coated with a hydrogel layer by pipetting melted agarose (1% low-melt agarose, Sigma) onto their surfaces. For single DIB experiments, each electrode was plugged into a female crimp-terminal, which was attached to a micromanipulator (Narishige, NMN-21). The other end of the female crimp-terminal (RS Components) was soldered to a cable that terminated with a male crimp, which was connected to the headstage of the amplifier.

### Preparation of Stocks of DPhPC in Oil

1,2-Diphytanoyl-sn-glycero-phosphatidylcholine (DPhPC) (Avanti, 4ME 16:0 PC) was dissolved in pentane, aliquoted into glass vials (7 mL, Supelco) and then dried with a stream of nitrogen gas. The dried films were dissolved at the requisite concentrations in filtered (0.22 μm Millex GP filters, Millipore) hexadecane (Sigma) and silicone oil (AR20, Sigma) mixed in a 1:1 ratio.

### Preparation of DPhPC and DPPE-mPEG2000 Lipid in Oil Stocks

DPhPC (Avanti, 4ME 16:0 PC) and DPPE-mPEG2000 (Avanti, 16:0 PEG2000 PE) were weighed out, dissolved in chloroform and combined in a glass vial. The solvent was removed in a stream of nitrogen gas and the residue was held under vaccum for >3 h, before storage at −20 °C. The dried films contained 2 μmol of total lipid with 2.5 mole% of DPPE-mPEG2000. Before use, dried films were dissolved in hexadecane (1 mL, Sigma), and then silicone oil (1 mL, AR20, Sigma) was added to give a total lipid concentration of 1 mM.

### Electrophysiology Chambers and Arrays

The chambers and arrays used throughout were manufactured by micromilling of poly(methyl methacrylate) (PMMA). Computer aided design (CAD) software (SolidWorks) was used to design the chambers in a format that was then executed by a subtractive computerized numerical control (CNC) machine (Roland Modela MDX-40A).

### General Electrical Recording Setup

Electrical recordings were conducted inside a Faraday cage, which contained a stage (Mechanical Workshop, University of Oxford) supporting the headstage of a conventional patch-clamp amplifier (Axopatch 200B, Axon Instruments) and two micromanipulators (Narishige, NMN-21) to guide the Ag/AgCl electrodes. The PMMA chamber, containing lipid-in-oil, was placed at the center of the stage.

### Fiber-Coupled LED

A 560 nm fiber-coupled LED (run at 100 mA) was used as an illumination source (Mightex, WFC-H7-0560). The LED was inserted at the top of the Faraday cage through an adaptor (Mechanical Workshop, University of Oxford) and aligned with the PMMA chamber. The light source was 4 cm above the Droplet-Hydrogel array. The power of the light from the LED that was incident on the droplet-hydrogel array was ∼1 mW (PM100A Compact Power Meter Console, Thorlabs). The illumination protocol was controlled from a computer by using the manufacturer’s software (Mightex).

### General Electrical Recording

A current signal was recorded with an amplifier (Axopatch 200B, Axon Instruments), connected to a digitizer (Digidata 1440 A, Axon Instruments), operating in gap-free acquisition mode at a sampling frequency of 10 kHz, and using a 2 kHz filter and five-fold gain. Data were analysed using Clampfit (version 10.3, Axon Instruments) by post-filtering with a 10 Hz low-pass filter. All electrical recordings were conducted at 22.0 ± 1.5 °C.

### General DIB Formation for Electrophysiology

Using micromanipulators (Narishige, NMN-21) and a stereo-microscope (Nikon, SMZ660), two hydrogel-coated electrodes were placed inside a micromilled square chamber with sides of length 5 mm and a depth of 3 mm. In this chamber, a 200 nL droplet was pipetted onto each of the two electrodes in the lipid-in-oil solution. Once a droplet had spontaneously adhered to the hydrogel coating, the electrode was lifted off the bottom surface of the chamber. As a result, the droplet hung on the electrode tip. After 5 min of incubation, the two droplets, one on each electrode, were gently brought together and spontaneously formed a droplet interface bilayer (DIB). To confirm that a bilayer had been formed, the capacitance was measured by applying a triangular voltage waveform (±15 mV, 30 ms peak-to-peak). A capacitance of ≥20 pF was taken to demonstrate that a bilayer had been formed.

### DHB Bio-pixel Recording

A PMMA mold (5 mm^2^, 3 mm depth) made with the CNC machine was used to form an agarose shape. A ground electrode (cis) was inserted into the hydrogel, while the working electrode (trans) was inserted into a 200 nL bR droplet, both in the same 14 mM DPhPC lipid-in-oil solution. After 20 min of incubation, the monolayer-coated hydrogel and droplet were gently brought together to form a droplet-hydrogel bilayer (DHB). To confirm that a bilayer had been formed, the capacitance was measured by applying a triangular voltage waveform (±15 mV, 30 ms peak-to-peak). A capacitance of ≥20 pF was taken to demonstrate that a bilayer had been formed. DHB bio-pixel formation was confirmed by the appearance of a current in the range of 1 to 15 pA, when the device was illuminated as described in ‘Fiber-Coupled LED’.

### DIB Bio-pixel Formation

DIB bio-pixels were formed in hexadecane:silicone oil 1:1 containing 7 mM DPhPC. The recording electrode (trans) was placed in the 200 nL bR-containing droplet and the ground electrode (cis) in the 200 nL Buffer A containing droplet. An active bio-pixel generated a positive steady-state current under illumination in the range of 1 to 15 pA.

### Electrical Response of Buffer A DIBs

Bare Ag/AgCl electrodes were placed in a DPhPC-containing oil solution (hexadecane:silicone oil 1:1, 7 mM lipid) in the Faraday cage. The current output was measured with and without illumination. Buffer A droplets (200 nL) were then pipetted onto the electrodes and the current recorded with and without illumination, prior to DIB formation. The two droplets were then brought into contact and a bilayer was formed, and the current was again recorded with and without illumination.

### Effect of DIB Area on the Current Profile Produced by bR

Bio-pixels were formed from two droplets in 7 mM DPhPC in hexadecane:silicone oil (1:1). The bilayer area was altered by pulling the droplets apart or pushing the droplets together with a micromanipulator (Narishige, NMN-21). The pixels were illuminated and the current profiles recorded.

### Direction of Insertion of bR in the Pixels

A DIB bio-pixel was formed in 7 mM DPhPC in 1:1 hexadecane:silicone oil. Initially, the droplet pair had bR in the trans droplet and Buffer A in the cis droplet. Light pulses of various durations were made. Then the Faraday cage was opened and the droplets separated before the configuration of the electrodes was changed to bR/cis and Buffer A/trans. The DIB was reformed and the light pulses repeated.

### 4 × 4 PMMA Device

The 4 × 4 device was built from two micromilled plastic parts (Fig. S5). Part 1 had four equally spaced holes on each of its sides and a central cavity with a rectangular cut-through (Fig. S5a,b and d). At the bottom, it had four rectangular bases at the edges (Fig. S5b–d). Part 2 had a rectangular cavity with a 4 × 4 array of equally spaced hole-containing pillars (Fig. S5e–h). The parts were then glued together and the electrical components integrated (Fig. S6). A gold over nickel-plated female crimp (RS Components, 446–800) was glued (Araldite Rapid, SLS) into each of the circular holes. 16 individual Ag/AgCl wire electrodes were soldered onto each female crimp and glued to the bottom of the PMMA device with the ends of the wires emerging from each of the holes in part 2. From the top, the female crimp was then soldered to individual cables (RS Components) 30 cm long and ~2 mm wide. The other end of each of these cables was soldered to a ring terminal (RS Components), used to connect to screw terminals on the multichannel amplifier’s electrode probe holders (Terrapin, Tecella LLC).

### Multichannel Electrical Recordings

Electrical recordings for the droplet-hydrogel arrays were conducted with a multichannel amplifier (Triton^+^, Tecella LLC) at 22.0 ± 1.5°C. Electrodes were connected to the amplifier via two 8-channel electrode probe holders (Terrapin, Tecella LLC), with each channel ending in a screw-terminal. The probe holders were contained in a Faraday cage and the 16 screw-terminals were each connected to one of the 16 electrodes in the 4 × 4 PMMA device, for individual recording from each electrode.

With the aid of a stereo-microscope (Nikon, SMZ660), a droplet was pipetted onto each of the 16 hydrogel-coated (1% w/v low-gelling agarose, Sigma) Ag/AgCl wire electrodes in the PMMA array. (The electrodes were formed and treated as described in ‘Preparation of Ag/AgCl Electrodes’).

The current signal was obtained by gap-free acquisition with a feedback resistor of 1 GΩ. Data were acquired at 20 kHz with a 5 kHz filter through the ‘TecellaLab v0.90 type 2′ software.

### Recognition of Light Shapes with a Prototype Light-Activatable DIB 4 × 4 Array

The PMMA device was filled with 200 μL of the lipid-in-oil solution (DPhPC and DPPE-mPEG2000 in 1:1 hexadecane:silicone oil). bR-containing droplets (100 nL) were placed on the end of each of the 16 electrodes (trans) and a droplet containing Buffer A was formed on the end of the moving electrode (cis) (Fig. S4). The moving droplet was guided through the array, forming a bilayer (and hence a bio-pixel) with each bR-containing droplet in turn.

The system was illuminated with the LED as described in ‘Fiber-Coupled LED’. The steady state current signals for each bio-pixel were plotted as histograms and a normal distribution fitted to each one. This allowed the discrimination of the ON and OFF pixels, by comparing the mean steady-state current with the cut-off value of 1 pA.

### 4 × 4 Array Lid

A removable lid (Fig. S7) was designed to fit inside the 4 × 4 array described previously. The lid had a 4 × 4 arrangement of 16 circular pillars. The pillars had the same center-to-center spacing as the PMMA 4 × 4 array. Two holes, each at opposite edges of the lid, were used to introduce the melted agarose.

### Design of 4 × 4 DHB Array with a Common Ground

A solution of 1.7% w/v low-gelling agarose (Sigma) in Buffer B was held at ~100 °C and then pipetted through the holes in the 4 × 4 array lid (previously coated with hexadecane: silicone oil 1:1). The solution was left to gel for 30 min at room temperature. The lid was then carefully removed, resulting in a hydrogel covering over the 4 × 4 array, with cavities that exposed the electrodes. A Ag/AgCl ground electrode was inserted into the hydrogel.

### Bio-Pixel Formation in the 4 × 4 DHB Array with a Common Ground

Droplets containing the suspension of bR were incubated in 14 mM DPhPC in 1:1 hexadecane: silicone oil for 30 min. The common ground hydrogel was incubated under the same conditions, by pipetting the lipid in oil solution into the device containing the hydrogel. A bR-containing droplet was pipetted into each cavity. Each droplet made contact with its respective electrode and formed a lateral bilayer with the surrounding hydrogel (Fig. 2). DHB bio-pixel formation was confirmed by the appearance of a current in the range of 1 to 15 pA, when the device was illuminated as described in ‘Fiber-Coupled LED’. No current was detected when no droplets or droplets containing only Buffer B were used.

### Photomasks

A 4 × 4 pixel mask, with the dimensions of the 4 × 4 PMMA device, was created in Illustrator (Adobe). Shapes were formed by colouring selected pixels black. The shapes were printed on A4 transparency sheets for mono printers (Xerox). Once printed, the 0% transparency areas of the shapes were made fully opaque with a fine black permanent market (Sharpie) and cropped as required. For grey-scale detection, shapes were printed with selected pixels of different transparency values, ranging from 0 to 100%.

### Recognition of Light Shapes with a 4 × 4 Droplet-Hydrogel Array

Photomasks were rested on top of the PMMA device and aligned with the 4 × 4 bio-pixel array. The Tetris-based photomasks were manually moved across the PMMA device. The system was illuminated with the LED. The mean steady-state current from each pixel was determined. We used the 1 pA cut-off to determine whether a pixel was ON or OFF.

### Data Analysis Software

The data stream of current measurements was saved to disk in Tecella’s tlc binary file type. The measurements were analysed and displayed using custom software written in LabVIEW. First, the tlc file was accessed by calling a custom C++ program, which acted as a wrapper to call data-reading functions provided by a Tecella API (TecellaTLC API v0.3). The data from each channel were then processed through a low pass Bessel filter (8th order, 5 Hz cut-off) to remove noise. For display purposes, the data were then re-sampled at 30 Hz. Next, a linear fit was performed for each channel independently using two user-specified portions of the data (typically corresponding to “OFF” states at the beginning and end of the measurement period). Any linear drift or offset was then removed by subtracting this fitted function from the filtered data. Finally, the results were displayed in two ways: a two-dimensional grid of graphs (plotting current against time), and a visualiser, on which each channel was represented by a “colour block” that switches from grey to green as the current transitions through an arbitrary threshold value defined by the user (1 pA in the present work). This threshold value defines “OFF” and “ON” states, and was also displayed on the graphs. A text overlay on the visualiser displays the current (in pA). Before being displayed on the visualiser, the data were further processed by applying an additional filter (median filter, rank 30), in order to make the resulting video easier to read by further reducing high frequency components.

### Tetris Video Processing

Tetris-based photomask shapes were positioned at nine different locations on the droplet-hydrogel array as described in ‘Recognition of Light Shapes with a 4 × 4 Droplet-Hydrogel Array’. For each position of a photomask, a video of the “visualiser” described in ‘Data Analysis Software’ was generated using our custom LabVIEW software. The visualiser displays a “colour block” and text overlay of the current (in pA) for each channel. The nine videos, each corresponding to one of the nine photomask positions (see Fig. S13), were then combined using Windows Movie Maker.

### Grey-scale detection

The bio-pixels were calibrated before and after the grey-scale images were examined by the array. To calibrate each bio-pixel, photomasks of 0, 25, 50, 75 and 100% transparency were placed on top of the entire array and then illuminated. Between the two calibrations, the grey-scale cross images were placed over the array and illuminated. The mean steady-state current value from each bio-pixel, and its standard deviation, was determined for both the calibrations and the cross images. Data from both calibrations were combined and plotted as output current against transparency value, for each bio-pixel (Fig. S14). Linear fits and their corresponding linear equations were acquired from these plots for each bio-pixel. Using these linear equations, the transparency value of each bio-pixel was calculated from the current output when the grey-scale photomask was held over the array. These calculated transparency values were compared to the actual transparency values of the photomask (Tables S1–3).

## Additional Information

**How to cite this article**: Restrepo Schild, V. *et al*. Light-Patterned Current Generation in a Droplet Bilayer Array. *Sci. Rep.* **7**, 46585; doi: 10.1038/srep46585 (2017).

**Publisher's note:** Springer Nature remains neutral with regard to jurisdictional claims in published maps and institutional affiliations.

## Supplementary Material

Supplementary Information

Supplementary Video
