# Supplementary material for: Light-Patterned Current Generation in a Droplet Bilayer Array
Source: Sci Rep. 2017 Apr 18;7:46585. doi: 10.1038/srep46585 (PMC5394532; doi:10.1038/srep46585)
Supplement: Supplementary Information [file srep46585-s1.pdf]

Light-Patterned Current Generation in a Droplet Bilayer Array

Vanessa Restrepo Schild, Michael J. Booth, Stuart J. Box, Sam N. Olof, Kozhinjampara R Mahendran, Hagan Bayley*

Chemistry Research Laboratory, University of Oxford, 12 Mansfield Road, Oxford, OX1 3TA, UK.
*hagan.bayley@chem.ox.ac.uk

Figure S1: A DIB bio-pixel generates a current under illumination.

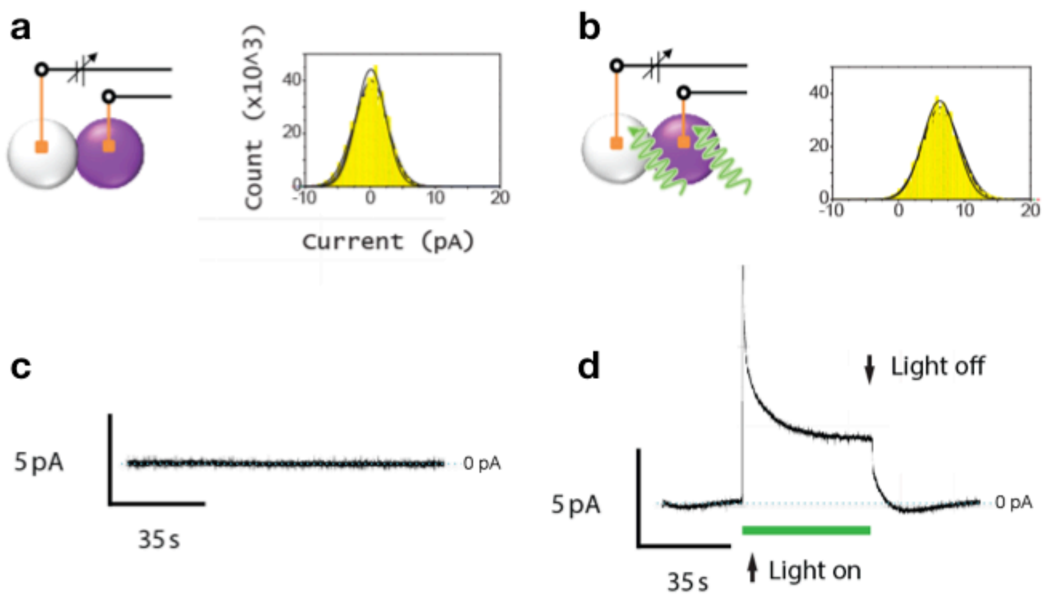

a, Schematic of a droplet-interface-bilayer (DIB) pixel. A DIB is formed from a buffer-containing (white) and bR-containing (purple) droplet, each connected to an electrode. The ground electrode was inserted in the buffer droplet. A histogram illustrated the baseline noise centered about 0 pA. **b**, The bio-pixel was illuminated and a ~5 pA increase in the steady-state current was observed. **c**, Filtered (10 kHz low pass filter) current trace of a DIB bio-pixel without illumination. **d**, Filtered current trace of an illuminated DIB bio-pixel showed the bR-current waveform. Five different measurements of the each DIB are included in the histograms shown in (a) and (b), and Gaussian fits (black lines) are shown for each.

Figure S2: The majority of bR protein inserts N-terminus first

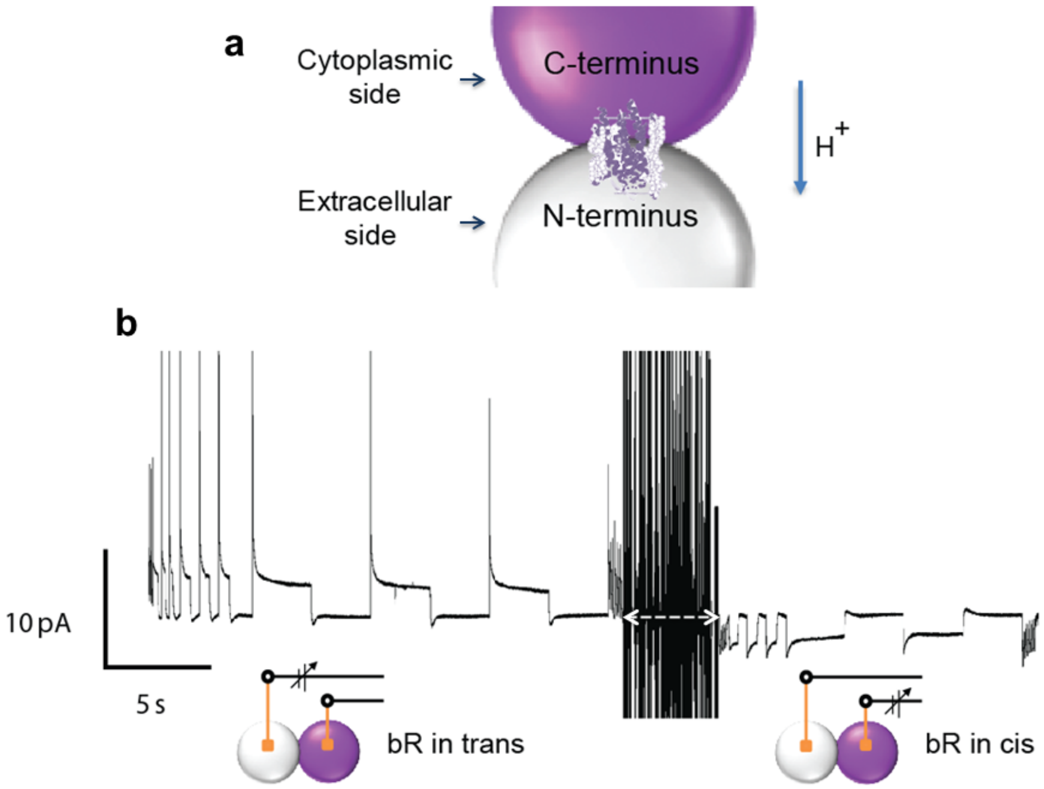

a, Schematic of the insertion of bR into a DIB. Proton movement in bR is from the C-terminal side to the N-terminal side. In H. salinarum, the C-terminus of bR is in the cytoplasm and protons are pumped out of the cell. **b**, Current trace of bR pumping activity during sequential light pulses. The recording electrode (trans) is in the droplet-containing bR (purple), while the ground electrode (cis) is in the buffer-containing droplet (white). A positive current was recorded, with the protons moving into the buffer droplet. The configuration of the electrodes was then reversed (white dashed line) and a negative current was generated upon illumination. This indicates that the C-terminus of the bR must be in the bR droplet and the N-terminus in the buffer droplet.

Figure S3: No current is observed from an illuminated DIB bio-pixel without bR.

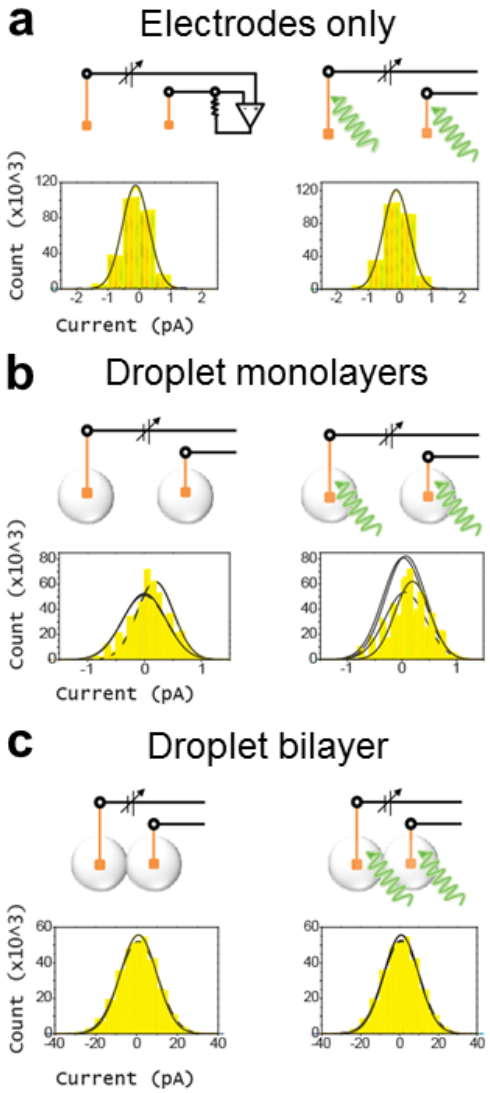

a, Bare Ag/AgCl electrodes were placed in 1:1 hexadecane:silicone oil containing DPhPC in the Faraday cage. The current output was measured with and without LED illumination (560 nm) and the baseline remained at 0 pA, indicating that the illumination had no measureable effect on the electrodes. **b**, Single Buffer A droplets were placed on each electrode. Illumination was performed as in (a) and the baseline remained at 0 pA. **c**, The two droplets were brought into contact and a bilayer was formed. The baseline noise increased as a result. Illumination was performed as in (a) and the baseline remained at 0 pA. Five different datasets are plotted on the histograms shown in (a), (b) and (c), and Gaussian fits are shown for each. The results indicate that illumination has no measurable effect on the current output of a bio-pixel in the absence of bR.

Figure S4: A light-activatable 4x4 DIB array

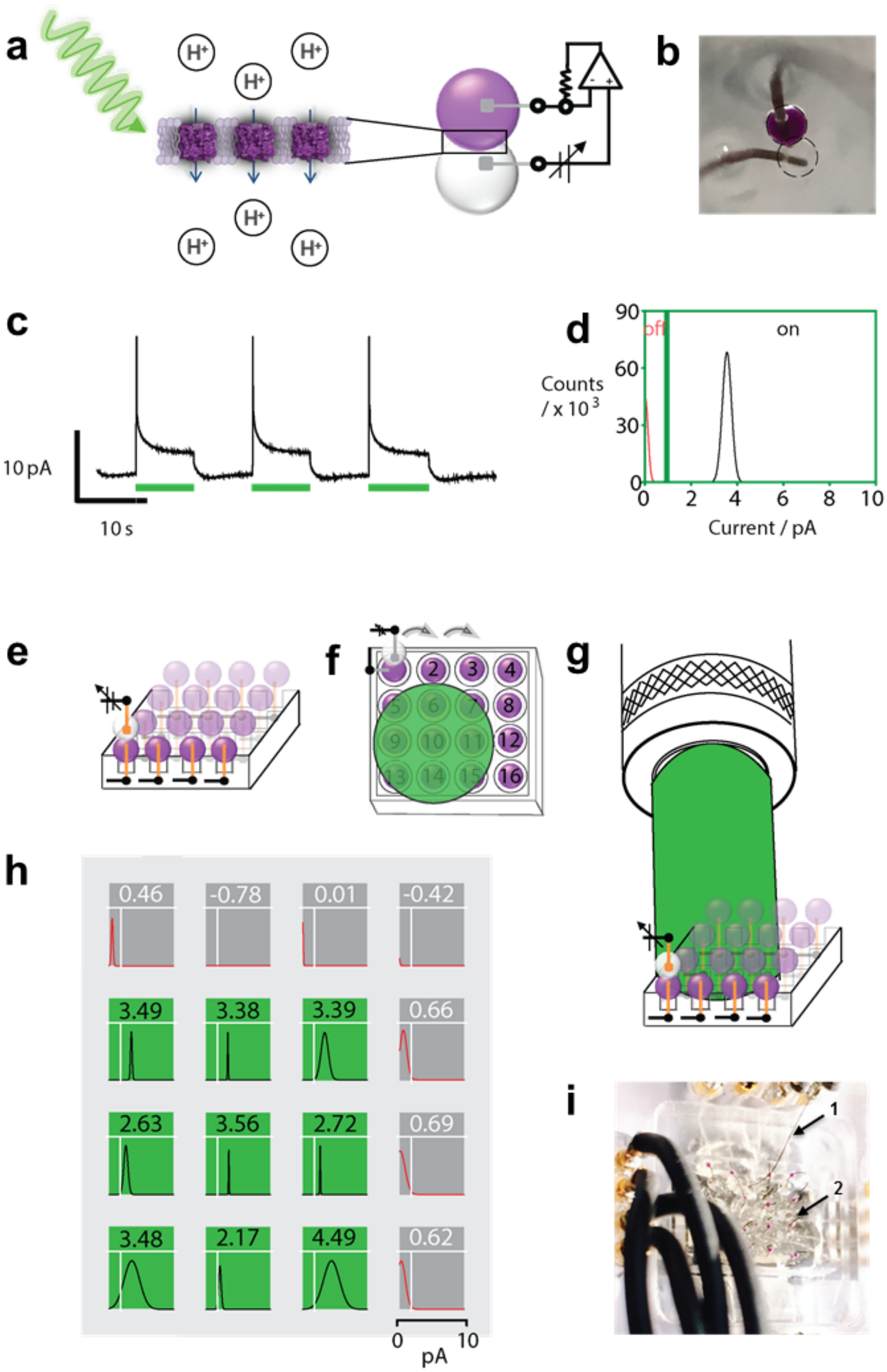

a, Schematic of a DIB bio-pixel. Green light activates vectorial H^+ pumping by bR generating a current across the membrane, which is measured through electrodes inserted into each droplet. **b**, Image of

a bio-pixel according to the schematic in (a). **c**, Ionic current is generated only upon illumination (green bars). **d**, Gaussian fits of the OFF (red) and ON (black) steady-state currents from (c). A pixel is considered ON when the steady-state current is above the cut-off of 1 pA (vertical green line). **e**, bR droplets were patterned onto a 4x4 array of recording electrodes, while a single buffer droplet was suspended on a moving agarose-coated electrode. The moving droplet was guided through the array, forming a DIB pixel with each bR droplet in turn. **f**, Schematic of a 4x4 droplet array, with a 3x3 section illuminated from above. **g**, The fiber-coupled LED (560 nm) was held perpendicular to the array. **h**, Identification of the illuminated DIB pixels from steady-state current distributions. When the steady state current is above the 1 pA cut-off (white dashed line), the pixel is green; and when below the cut-off, it is grey. **i**, An image of the 4x4 light-activatable DIB array showing (1) the moving electrode and (2) a bR-containing droplet.

Figure S5: Parts of the 4x4 PMMA device

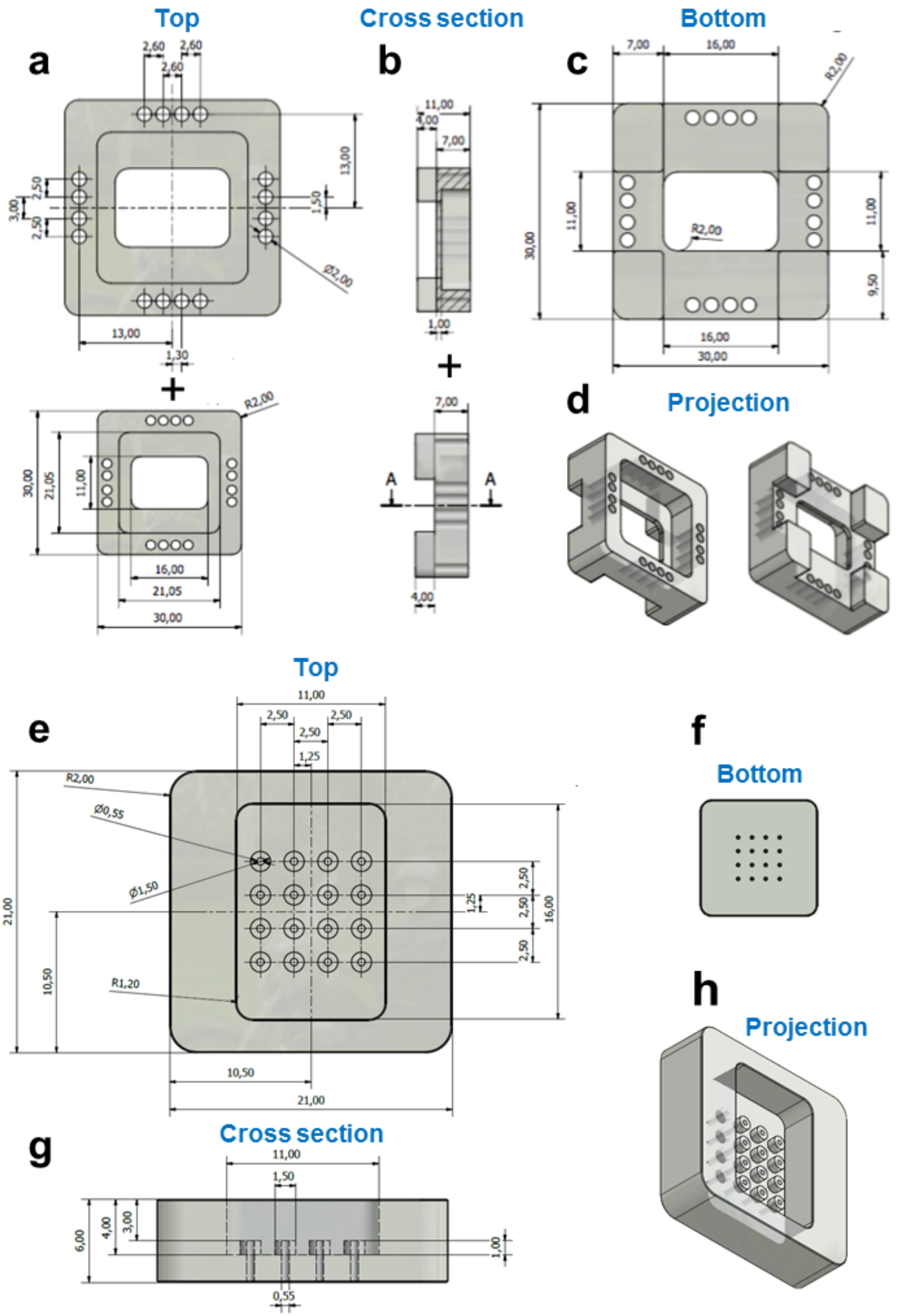

a, Schematic top-view of part 1 of the PMMA device. It has four equally spaced holes on each of its sides and a central cavity with a rectangular cut-through. **b**, Schematic cross-sections of part 1, showing the internal cavity and the holes on each side. **c**, Schematic bottom-view of part 1 with four rectangular feet at the edges. **d**, Schematic projection-view of part 1 displaying the holes on each side, the internal cavity, the cut-through, and the feet. **e**, Schematic top-view of part 2 of the PMMA device. In the center there is a rectangular cavity with a 4x4 array of equally spaced hole-containing pillars. **f**, Schematic bottom-view of the part 2 exposing the 4x4 array of holes. **g**, Schematic cross-section of part 2, showing the rectangular cavity, the pillars and holes. **h**, Schematic projection-view of part 2.

Figure S6: The 4x4 PMMA device with its electrical wiring

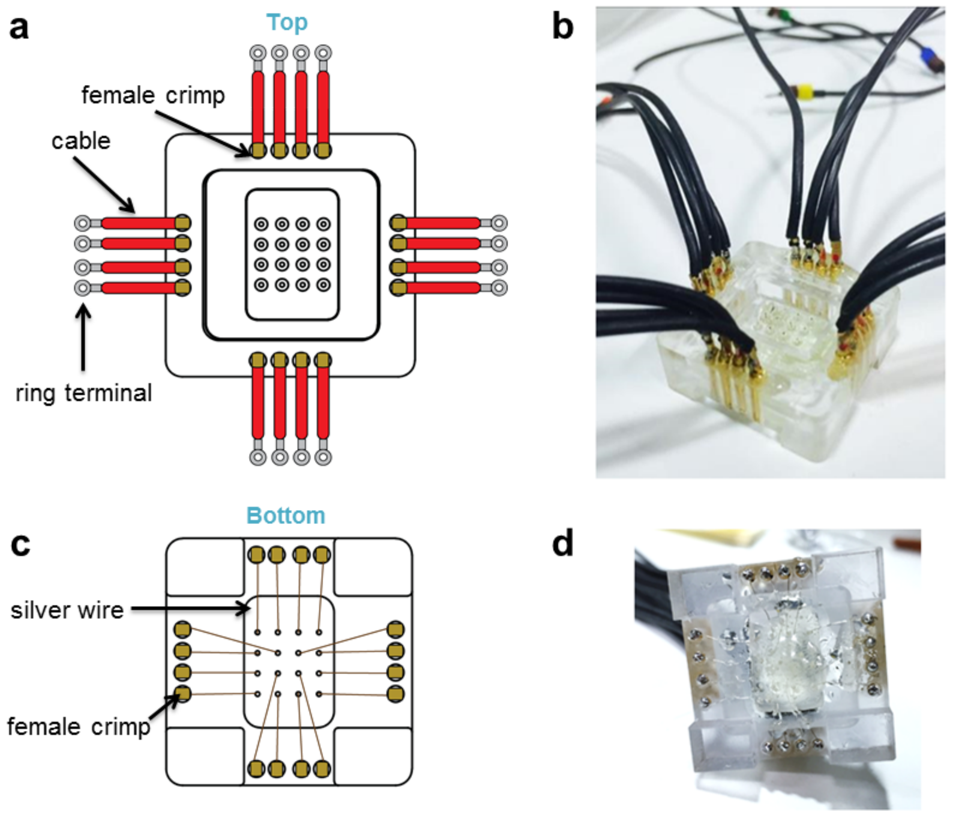

a, Schematic top-view of the PMMA device formed from the assembly of part 1 and 2. A gold-plated female crimp is glued into all 16 outside holes of part 1, to allow cables with ring terminals to interface the PMMA device to the multichannel amplifier. **b**, Image (projection-view from the top) of the PMMA device. **c**, Schematic bottom-view of the PMMA device. The gold-plated crimps inserted in part 1 are soldered to separate silver wires that enter the 16 holes of part 2 and are glued in place. **d**, Image (projection-view from the bottom) of the PMMA device.

Figure S7: Formation of the synbio hydrogel retina

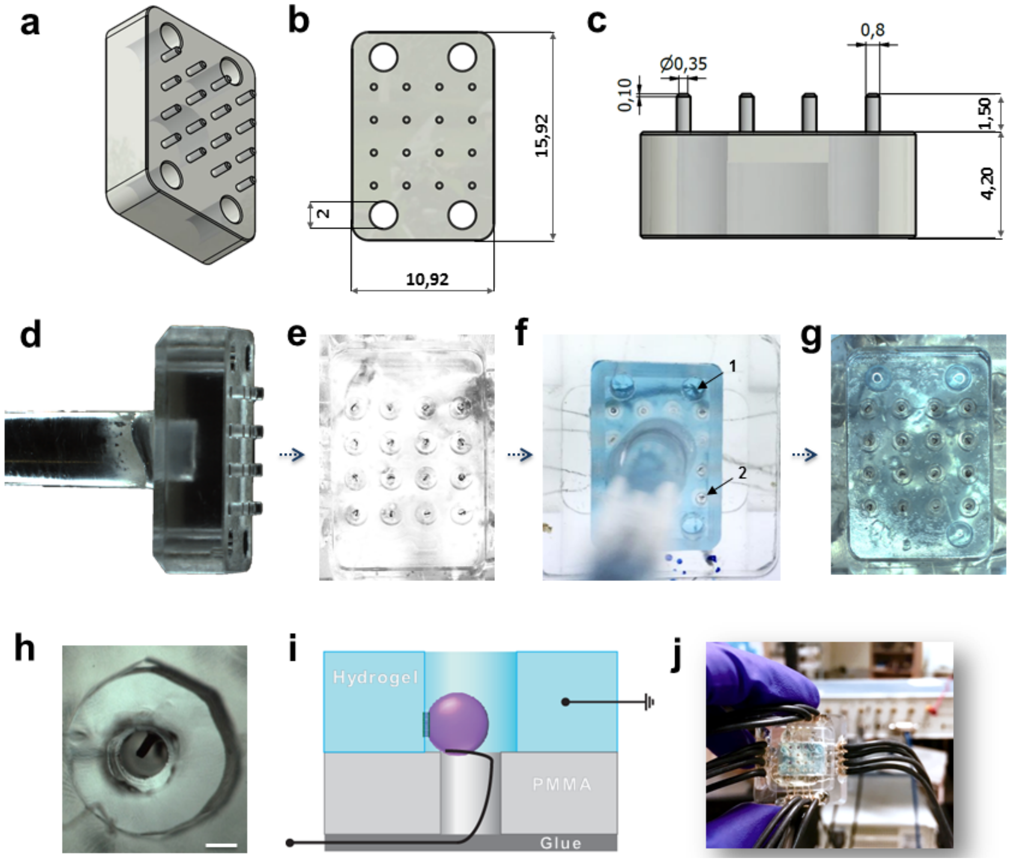

a, Projection-view of the lid made of PMMA with pillars that aligns with the wells of the PMMA device. **b**, Top-view of the lid. **c**, Side-view of the lid. **d**, Image of the lid made of PMMA with holder glued to its back. **e**, PMMA device with electrodes present in each of the 16 wells. **f**, The lid fits inside the PMMA device and melted agarose can be added through the opening (1). A hydrogel-free cavity is formed above each of the 16 hole-containing pillars in the PMMA device (2). **g**, When the lid is removed an agarose hydrogel structure with sixteen cavities for bio-pixels is formed. **h**, A hydrogel bio-pixel cavity, with an electrode in the middle of the well. Scale bar: 330 µm. **i**, Schematic cross-section of bio-pixel formation in the hydrogel cavity. The PMMA wells shown are the hole-containing pillars of the PMMA device. **j**, The Droplet-Hydrogel array outside the Faraday cage.

Figure S8: Electrical recording analysis and visualization software

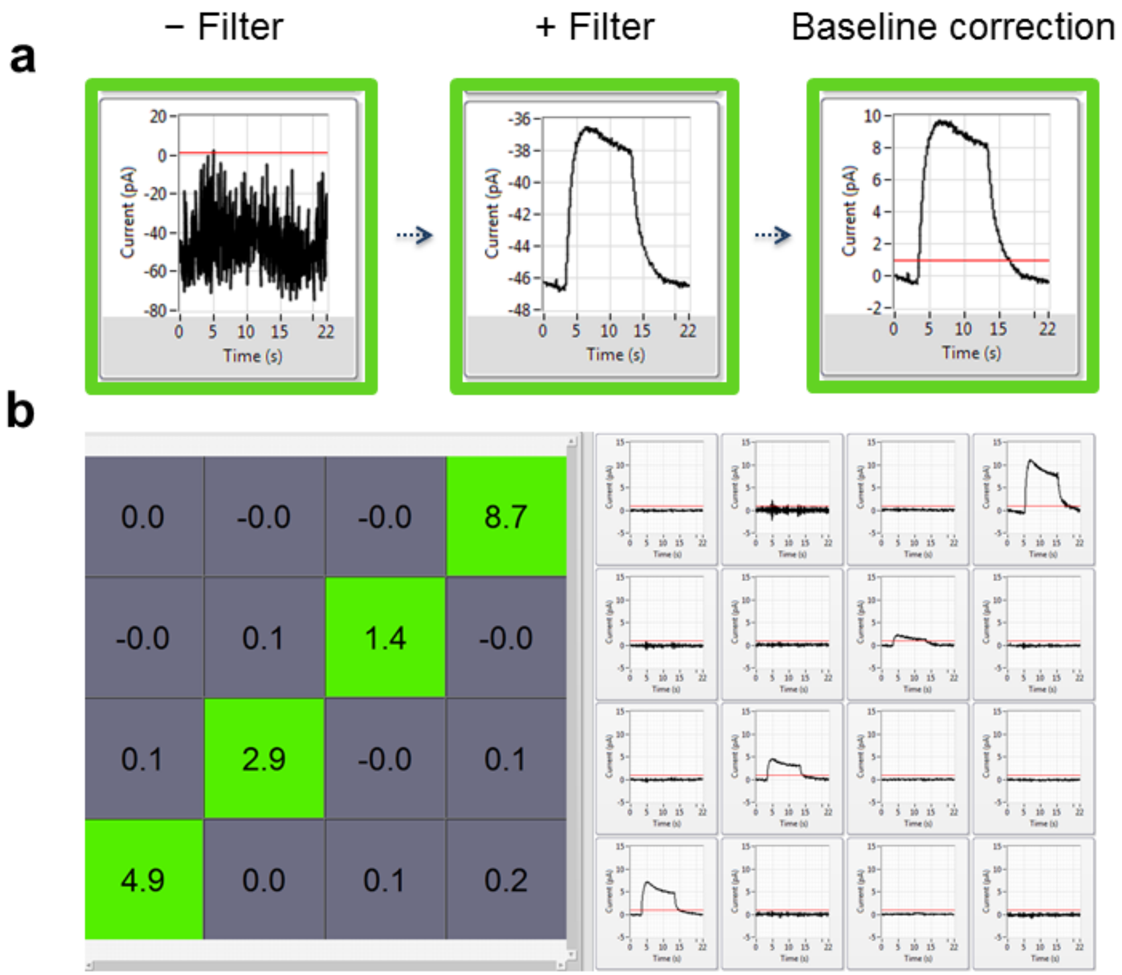

a, The custom software read a raw current trace, applied an additional filter (after the 5 kHz recording filter, see Methods) and corrected the baseline. The red line is the 1 pA cut-off. Baseline correction was required as the multichannel amplifier does not balance the electrodes. **b**, The current at any time point was shown on a visualiser (left), which had a grey background and turns green if the current went above the cut-off. The current was also plotted against time (right) to show the current waveform for each bio-pixel.

Figure S9: The 4x4 Droplet-Hydrogel array only detects light when bR droplets are included

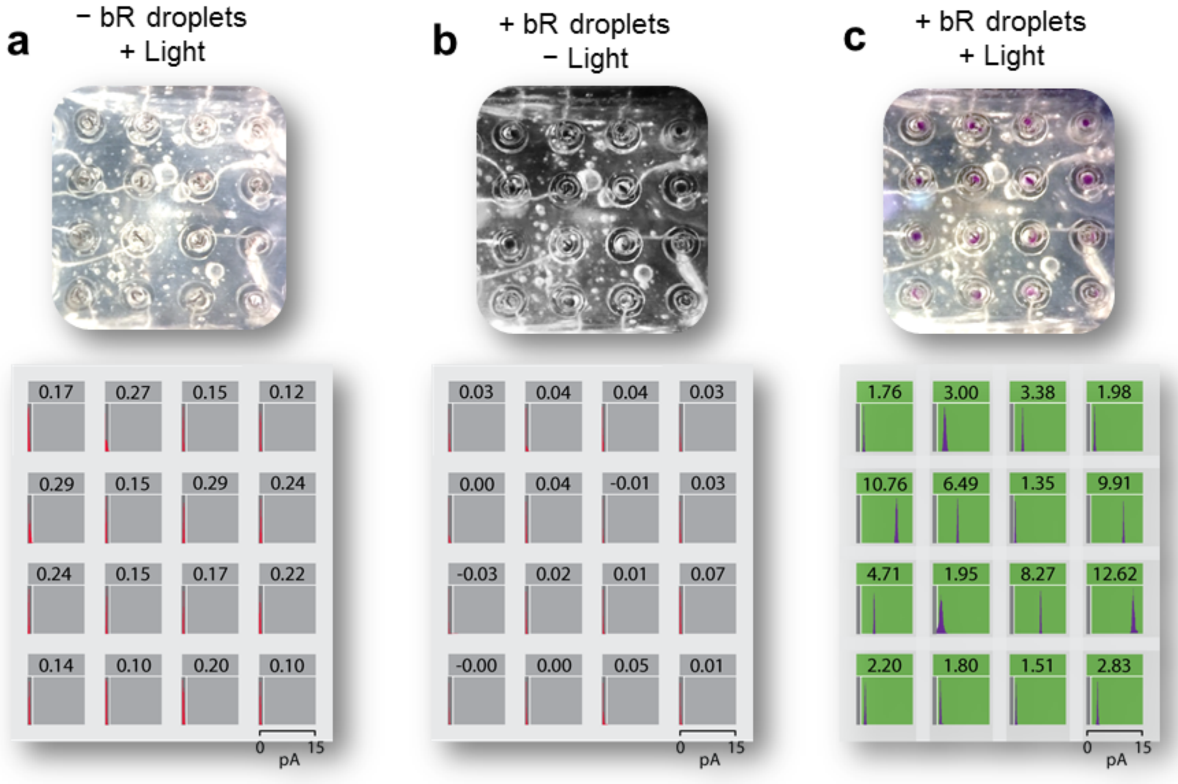

a, The hydrogel was formed with the hollow cavities but without any bR-containing droplets. Upon illumination, no current was detected from any electrode. **b**, When bR-containing droplets were included, no current was detected in the dark. **c**, When bR-containing droplets were included and the device was illuminated all 16 bio-pixels detected the light.

Figure S10: Capacitance modulates the current-profile of bR in DIBs

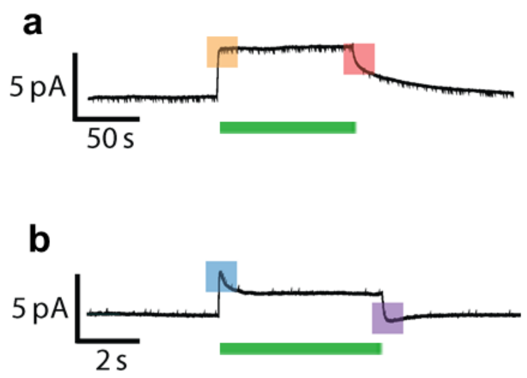

Two examples of how the bR current profile was modulated by bilayer capacitance. A large bilayer area resulted in a high capacitance and a small bilayer area in a low capacitance. **a**, A capacitance of ~800 pF generated undershoot signals when the protein was turned ON (orange box) and OFF (red box). Observed as a transitional current not exceeding the steady state (orange box) or the baseline (red box) current. **b**, A capacitance of ~30 pF generated overshoot signals when the protein was turned ON (blue box) and OFF (purple box). Observed as a transitional current exceeding the steady state (blue box) and the baseline (purple box) current.

Figure S11: Photomasks printed on transparency foil

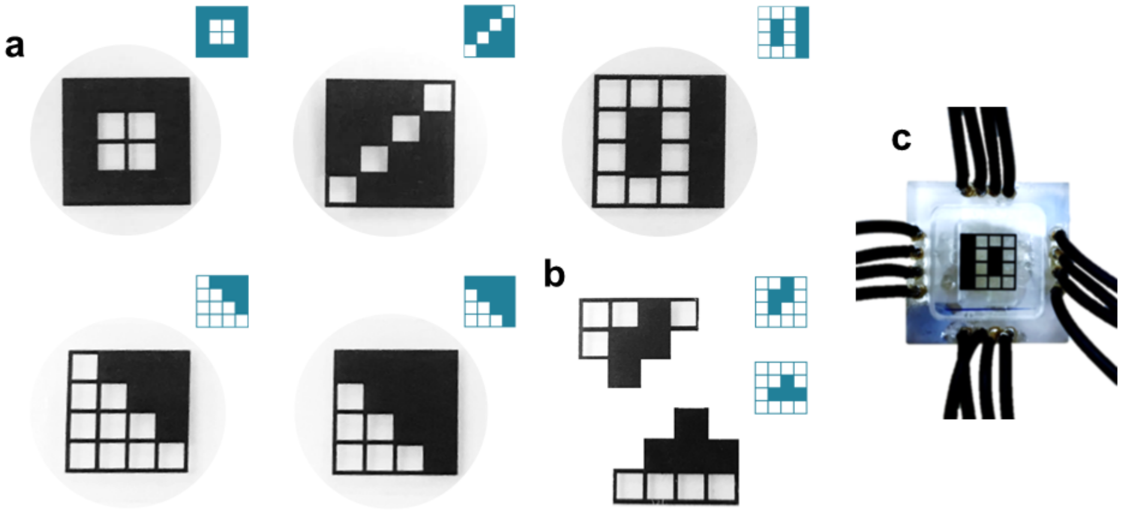

a, Image of the photomasks used for static shape detection. **b**, Image of the Tetris-based photomasks used for moving shape detection. **c**, A photomask on top of the PMMA device.

Figure S12: Static shape recognition with the Droplet-Hydrogel array

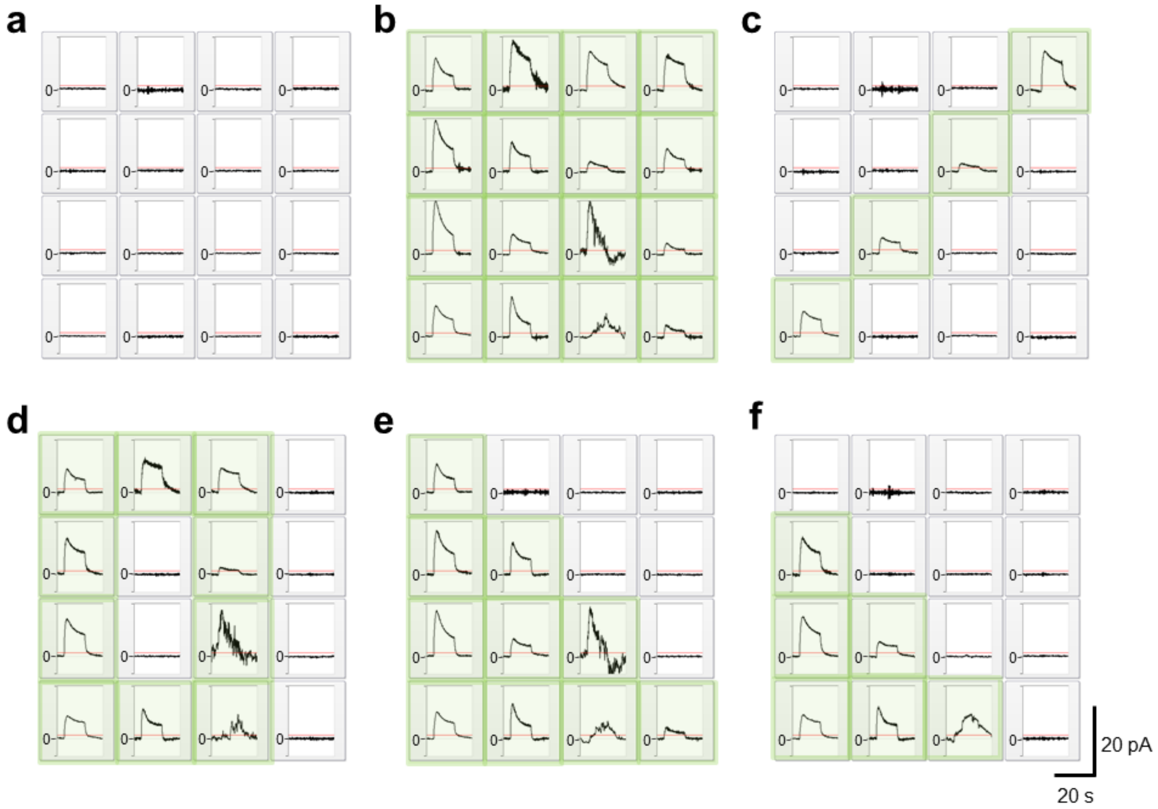

a, Current traces from the Droplet-Hydrogel array in the dark. No currents were detected above the 1 pA cut-off (red line). **b**, The whole Droplet-Hydrogel array was illuminated and a current waveform was detected in every bio-pixel. A green border and background was placed over each pixel where current is observed. **c**, The Droplet-Hydrogel array was illuminated through a photomask with a diagonal line. Current waveforms were detected in bio-pixels that correspond to the pattern of illumination. **d**, Current waveforms generated with a photomask of the letter O. **e**, Current waveforms generated with a large triangular photomask. **f**, Current waveforms generated with a small triangular photomask.

Figure S13: Detecting moving images with the Droplet-Hydrogel array

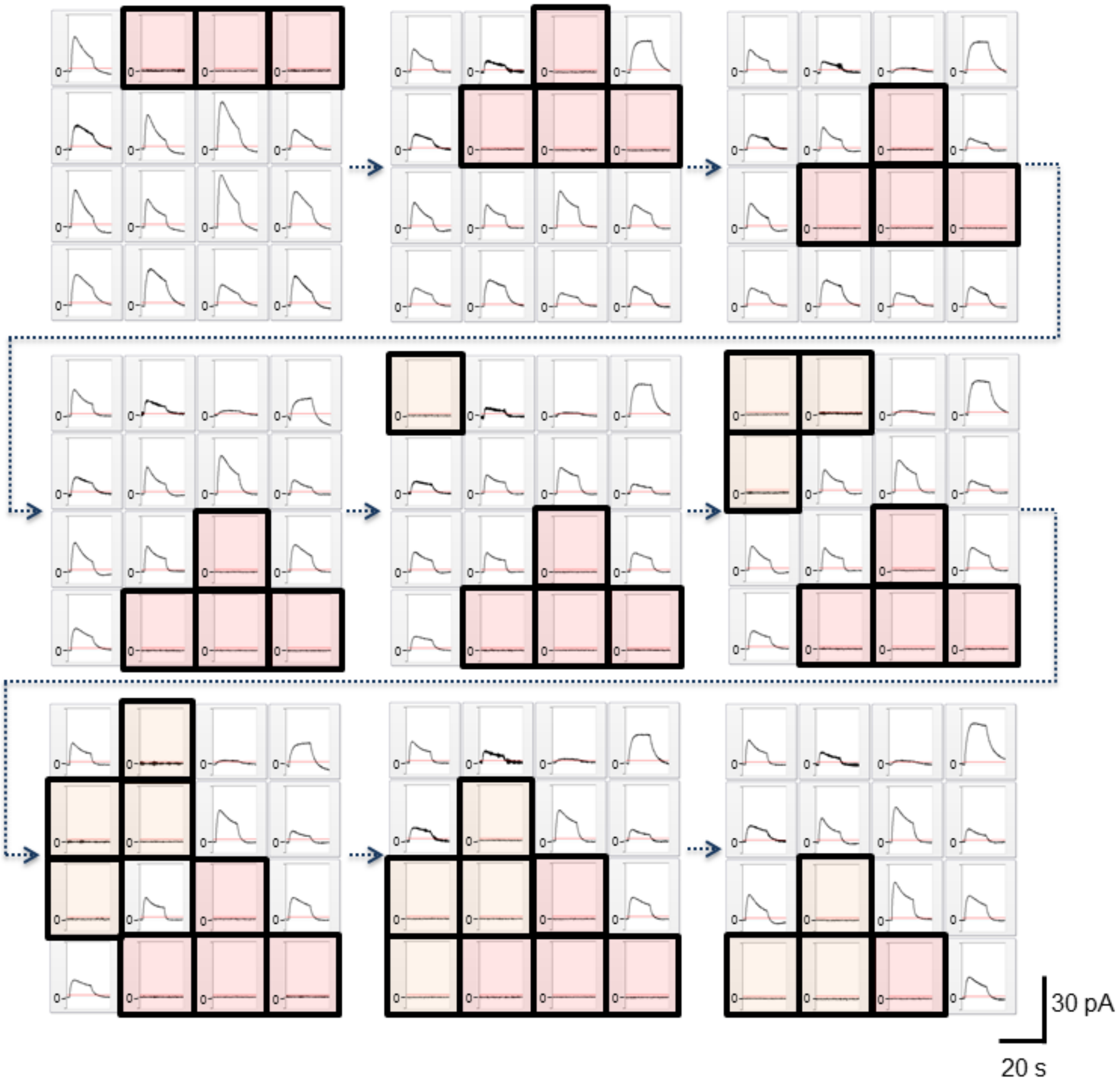

Current responses of the bio-pixels as Tetris-based shapes were manually moved down the face of the device. Each bio-pixel showed a current waveform when illuminated, apart from those that were blocked by the shapes. A black border surrounded each bio-pixel in which the current did not cross the 1 pA cut-off. The first Tetris shape was given a red background and the second shape a yellow background.

Figure S14: Calibration of a bio-pixel for grey-scale imaging

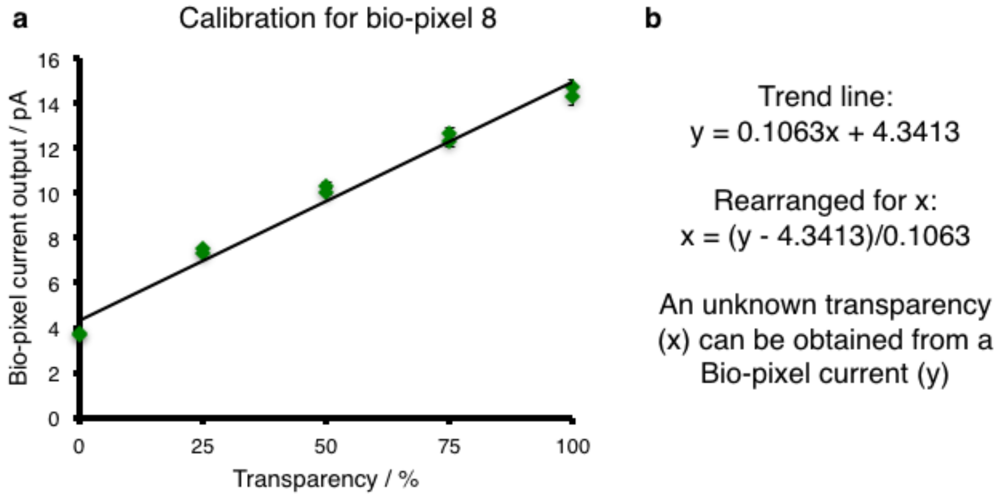

a, Calibration of bio-pixel 8 from the array. Bio-pixel current output was plotted against the corresponding photomask transparency values of 0, 20, 50, 75 and 100%. Current values for the 0% transparency are above the 1 pA cut-off as the inkjet printer cannot print the photomasks fully opaque. **b**, A linear fit was obtained from the calibration. The equation describing the line was rearranged to yield transparency values from currents produced by the array.

Table 1. Bio-pixel output and calculated transparencies from the top cross image in Figure 5.

Pixel	Actual Transparency	Bio-pixel Output		Calculated Transparency	
		Current / pA	SD	Transparency / %	SD
2	80	17.5	0.1	80.2	0.4
5	80	2.9	0.1	76.9	4.2
6	100	14.5	0.3	94.0	3.1
7	80	2.7	0.1	77.4	3.8
8	40	8.3	0.1	37.5	0.9
10	80	11.5	0.3	74.3	2.5
14	40	3.5	0.1	38.5	1.5

Table 2. Bio-pixel output and calculated transparencies from the middle cross image in Figure 5.

Pixel	Actual Transparency	Bio-pixel Output		Calculated Transparency	
		Current / pA	SD	Transparency / %	SD
2	60	14.7	0.0	61.8	0.3
5	60	2.6	0.1	62.9	2.8
6	100	14.6	0.3	94.9	3.1
7	60	2.5	0.1	63.1	3.0
8	20	6.1	0.0	16.5	0.3
10	60	9.5	0.2	55.3	1.6
14	20	2.7	0.1	17.3	1.8

Table 3. Bio-pixel output and calculated transparencies from the bottom cross image in Figure 5.

Pixel	Actual Transparency	Bio-pixel Output		Calculated Transparency	
		Current / pA	SD	Transparency / %	SD
2	60	14.8	0.0	62.1	0.3
5	60	2.6	0.1	63.3	2.8
6	20	6.8	0.0	20.9	0.3
7	60	2.5	0.1	65.4	3.0
8	100	15.3	0.4	103.1	3.6
10	60	9.7	0.2	57.2	1.6
14	100	6.0	0.1	99.4	2.3

Video S1: Detecting moving images with the droplet array.
Two Tetris-based shapes moving down the face of the droplet array one pixel at a time. When the bottom row was completed, both of the Tetris blocks were moved down together by one pixel. The visualiser displays a “colour block” and text overlay of the current (in pA) for each channel. The shapes were revealed by the currents in the active bio-pixels (green) in contrast to those in the inactive bio-pixels (grey).
